# Microspectroscopy of spectral biomarkers associated with human corneal stem cells

**Published:** 2010-03-06

**Authors:** Takahiro Nakamura, Jemma G. Kelly, Júlio Trevisan, Leanne J. Cooper, Adam J. Bentley, Paul L. Carmichael, Andrew D. Scott, Marine Cotte, Jean Susini, Pierre L. Martin-Hirsch, Shigeru Kinoshita, Nigel J. Fullwood, Francis L. Martin

**Affiliations:** 1 Department of Ophthalmology, Kyoto Perfectural University of Medicine, Kawaramachi Hirohoji, Kamigyo-ku, Kyoto 602-0841, Japan; 2 Centre for Biophotonics, Lancaster Environment Centre, Bailrigg, Lancaster University, Lancaster, UK; 3 School of Health and Medicine, Biomedical and Life Sciences, Lancaster University, Lancaster, UK; 4 Safety and Environmental Assurance Centre, Unilever Colworth Science Park, Bedfordshire, UK; 5 European Synchrotron Radiation Facility, Grenoble, France

## Abstract

**Purpose:**

Synchrotron-based radiation (SRS) Fourier-transform infrared (FTIR) microspectroscopy potentially provides novel biomarkers of the cell differentiation process. Because such imaging gives a “biochemical-cell fingerprint” through a cell-sized aperture, we set out to determine whether distinguishing chemical entities associated with putative stem cells (SCs), transit-amplifying (TA) cells, or terminally-differentiated (TD) cells could be identified in human corneal epithelium.

**Methods:**

Desiccated cryosections (10 μm thick) of cornea on barium fluoride infrared transparent windows were interrogated using SRS FTIR microspectroscopy. Infrared analysis was performed through the acquisition of point spectra or image maps.

**Results:**

Point spectra were subjected to principal component analysis (PCA) to identify distinguishing chemical entities. Spectral image maps to highlight SCs, TA cells, and TD cells of the cornea were then generated. Point spectrum analysis using PCA highlighted remarkable segregation between the three cell classes. Discriminating chemical entities were associated with several spectral differences over the DNA/RNA (1,425–900 cm^−1^) and protein/lipid (1,800–1480 cm^−1^) regions. Prominent biomarkers of SCs compared to TA cells and/or TD cells were 1,040 cm^−1^, 1,080 cm^−1^, 1,107 cm^−1^, 1,225 cm^−1^, 1,400 cm^−1^, 1,525 cm^−1^, 1,558 cm^−1^, and 1,728 cm^−1^. Chemical entities associated with DNA/RNA conformation (1,080 cm^−1^ and 1,225 cm^−1^) were associated with SCs, whereas protein/lipid biochemicals (1,558 cm^−1^ and 1,728 cm^−1^) most distinguished TA cells and TD cells.

**Conclusions:**

SRS FTIR microspectroscopy can be employed to identify differential spectral biomarkers of SCs, TA cells, and/or TD cells in human cornea. This nondestructive imaging technology is a novel approach to characterizing SCs in situ.

## Introduction

Compared to embryonic or induced pluripotent stem cells (SCs), adult SCs might be used in clinical applications with minimal ethical problems. However, their in situ location remains poorly understood, and the emphasis has been to find unique SC “biomarkers.” Such approaches include immunolabeling, which identifies only a few molecules or epitopes per sample and does not give an integrated cell fingerprint. Furthermore, such a SC marker for one tissue type may not translate to a different one. A superior approach is to interrogate the entire cell fingerprint, and this might be achieved using mid-infrared (IR) spectroscopy (e.g., Fourier-transform IR [FTIR]) spectroscopy. It has been shown that it is possible to apply FTIR spectroscopy to distinguish between SC, transit-amplifying (TA) cells, and terminally-differentiated (TD) cells in bovine cornea [1] and between SCs and TA cells in human cornea [2]. The putative SC locations in human intestine were also highlighted using this approach [3].

Adult SCs underlie the regenerative ability of tissues that undergo continuous turnover. They are slow-cycling cells with a capacity for prolonged self-renewal throughout adult life [4]. One of the more understood and possibly simpler SC systems is that of the adult corneal epithelium. First suggested by Davanger and Evensen [5], the epithelial cells of the corneal limbus are believed to be responsible for renewal of the corneal epithelium. It is now generally accepted that the SC population is localized to the basal layer in the limbus [6-9]. Damage or disease in the limbal region results in cell invasion from the conjunctiva [10], whereas grafting of cells from a healthy region of the limbus regenerates the epithelium.

Corneal SCs can divide asymmetrically to produce one daughter SC and one TA cell. These TA cells, which have only limited proliferative capacity, have been observed to migrate from the limbus to the cornea, forming a basal cell layer. TA cells in turn divide to produce TD cells, which are highly specialized and have no proliferative capacity [11]. The most superficial layers of the corneal epithelium consist of TD cells, which are shed by desquamation. However, there remains no definitive biomarker of corneal epithelial SCs, but a small number of molecules might be differentially expressed in comparison with TA cells and TD cells; these include the presence of the keratin isoform K15 and the transcription factor p63 and the absence of gap junction proteins [12]. Because of their unique ultrastructural appearance, transmission electron microscopy (TEM) is also useful for the in situ localization of SC, TA cells, and TD cells. As SCs in the limbus have a clearly defined location and are accessible for surgical intervention, the study of the corneal epithelium has helped considerably in our understanding of how adult SCs function. Additionally, because the cornea is partially immunologically privileged, there has been a rapid development of ex vivo SC expansion and transplantation techniques for ocular surface disorders [13-17].

Although FTIR spectroscopy has been used for decades, its general application to cell biology has only been generally appreciated in the last decade. It has been used to detect changes associated with Alzheimers disease, osteoporosis, and to distinguish between malignant and nonmalignant cells in several different tissues [18-21]. Other applications have included studies of cell cycle [22] and to discriminate between SCs, TA cells, and TD cells [1]. Cellular biomolecules absorb the mid-IR (λ=2–20 μm) to give rise to characteristic spectra providing unique information regarding structural and conformational changes [23-26]. Conventional bench-top FTIR spectrometers have a relatively dim thermal IR source, resulting in a relatively poor signal-to-noise ratio at a cellular spatial resolution. In contrast, synchrotrons provide a highly collimated beam of light that is orders of magnitudes more brilliant. Using the mid-IR portion of synchrotron-based radiation (SRS) at 10-μm spatial resolution, the signal-to-noise ratio is approximately 1,000 times greater than benchtop sources [18,27]. An individual IR spectrum of cellular material is complex, and in a typical experiment that can involve the acquisition of hundreds of spectra, it is difficult to identify important, and often subtle, differences between cell types. A readily applicable means of interrogating such data is the use of multivariate analysis, such as principal component analysis (PCA) [28]. Reduction of complex IR spectral datasets in scores plots allows one to compare their similarity or dissimilarity in an unsupervised fashion based on how they cluster or segregate in the orientation that they are viewed; this then allows one to identify the loadings (i.e., wavenumbers) that are responsible for separation in a given direction of particular clusters [28].

In situ nondestructive biomarkers of SCs remain elusive. In this study we set out to investigate whether SRS FTIR microspectroscopy could be applied to identify spectral markers segregating the putative SCs, TA cells, and TD cells of adult human corneal epithelium. If such novel biomarkers were identifiable, corresponding sections could then be mapped to localize their spatial location. Spectral maps isolating the limbal region of human cornea would potentially provide a novel method for SC localization and characterization.

## Methods

### Tissue samples

Corneal specimens (normal healthy human corneas from both male and female donors with an average age of 65) were obtained from the Northwest Lions Eye Bank, Seattle, WA. The eyes were harvested within 12 h of death, and the corneas were placed in Optisol corneal preservation medium (Chiron Vision, Claremont , CA) and stored at 4 °C for up to 5 days before use.

### Immunohistochemistry

Based on a previously described method [29,30], 8-μm-thick cryostat sections were placed on gelatin-coated slides, air dried, and rehydrated in PBS (Phosphate buffered saline, 0.01 M phosphate buffer, 0.138 M NaCl, 0.0027 M KCl, pH 7.4) at room temperature (RT; 24 °C) for 15 min. To block nonspecific binding, sections were incubated with 2% bovine serum albumin (RT, 30 min; Sigma-Aldrich, St Louis, MO). Sections were then incubated (RT, 1 h) with the primary antibody (K15 [rabbit polyclonal antibody; Abcam, Cambridge, UK]) and laminin-5 (mouse monoclonal antibody; Chemicon, Temecula, CA) and washed three times in PBS containing 0.15% Triton X-100 for 15 min. Control incubations were incubated with appropriate normal mouse and rabbit immunoglobulin (IgG; Dako, Tokyo, Japan) at the same concentration as the primary antibody. After staining with the primary antibody, sections were incubated with appropriate secondary antibodies (RT, 1 h), Alexa Fluor 488 conjugated antirabbit IgG antibody and Alexa Fluor 594 conjugated antimouse IgG antibody (Molecular Probes Inc., Eugene, OR), washed several times with PBS, coverslipped using antifading mounting medium containing DAPI (4',6-diamidino-2-phenylindole; Vectashield; Vector, Burlingame, CA), and examined under a confocal microscope (TCS-SP2; Leica, Tokyo, Japan).

### Transmission electron microscopy

Specimens were fixed in 2.5% glutaraldehyde and post fixed in 2% osmium tetroxide. Prior to TEM, specimens were washed three times with PBS before being passed through a graded ethanol series (50%, 70%, 80%, 90%, 95%, and 100%) and embedded in epoxy resin. Ultrathin (70 nm) sections were collected on copper grids, stained, and examined using a transmission electron microscope (JEM 1010; JEOL, Tokyo, Japan).

### Data collection

FTIR data from cryosections of human cornea (10-μm thick on barium fluoride windows) were obtained on the ID21 beamline at the European Synchrotron Radiation Facility (ESRF), Grenoble, France. A Nexus-FTIR spectrophotometer (Thermo Scientific Inc., Waltham, MA) coupled to a Nicolet Continuum microscope and mercury cadmium telluride detector (Thermo  Scientific) cooled with liquid nitrogen, with a measuring range of 650–4,000 cm^−1^, was used. Spectral collection of IR spectra was made in transmission mode (4 cm^−1^ resolution, 8 μm×8 μm, co-added for 256 scans), and spectra were converted to absorbance using Thermo Omnic 7.1 software (Thermo Scientific Inc.). Raw spectra were processed by 13-point smoothing, baseline corrected, and normalized to the amide I (1,650 cm^−1^) absorbance peak, using Bruker OPUS software (Bruker Optics Inc., Billerica, MA).

Derived IR spectra (1,800–900 cm^−1^) were composed of several distinct peaks, which are associated with certain biochemical entities. They can be roughly divided into the protein region (1,800–1,480 cm^−1^) and the DNA/RNA region (1,425–900 cm^−1^). SC IR spectra were collected from the basal cell layer in the limbal region, which consisted of small primitive cells that were poorly differentiated and whose appearance was consistent with that of limbal SCs. TA cell IR spectra were collected from the basal cell layer in the cornea, approximately 3 mm away from the limbus. TD cell IR spectra were collected from the superficial cells, again about 3 mm from the limbus.

### Spectral mapping

Synchrotron FTIR spectral image maps of human cornea and limbus samples (in which image contrast is determined by the absorbance intensity at a chosen wavenumber) were obtained in transmission mode. At ESRF an aperture of 8 μm×8 μm was used with a step size of 8 μm, allowing maps composed of pixels (256 co-additions) at an 8 μm×8 μm resolution to be generated within an acquisition time of about 6 h. Spectral maps were baseline corrected, and two-dimensional (2-D) maps were processed with either linear or spline smoothing, using Thermo Omnic 7.1 software. During SRS FTIR microspectroscopy analyses, a new background was taken every 30 min to correct for atmospheric alterations or changes in beam current.

### Computational analysis

The primary constituents of the protein region are the peaks amide I (centered at 1,650 cm^−1^) and amide II (centered at 1,540 cm^−1^). PCA was conducted on spectra, using the Pirouette software package (Infometrix Inc., Woodinville, WA). After baseline correction and normalization, the spectra were processed as raw spectra. Nine principal components (PCs) were selected for analysis, and loading curves for each PC were plotted for each sample. These loading curves allowed the influence of specific spectral features on each PC to be identified. Scores plots (2-D) of each PC pair were then plotted for each sample, and by combining the clustering evident in these figures with the analysis of the loadings curves, the most appropriate three PCs were selected for the 3-D cluster analysis.

Per spectral feature, histograms were also computed to visualize differences in relative absorbance distributions for each of the three different cell types (i.e., SCs versus TA cells versus TD cells). These histograms were computed as distribution percentages along 100 equal-length subintervals of the whole absorbance range [0, 2]. All one-wavenumber histograms were set together in a 3-D form to facilitate comparative visualization of cell-specific profiles.

## Results

### Conventional verification of putative regions

**Figure 1:**
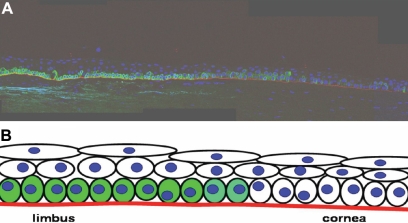
Immunolabeling for the presence or absence of stem cells (SC) in human cornea. **A**: Section of limbal and peripheral cornea immunolabeled with a green fluorescent marker (Keratin 15) for corneal SCs. The cell nuclei have been stained with DAPI (4',6-diamidino-2-phenylindole; blue) and the basement membrane immunolabeled for laminin (red). **B**: Schematic diagram of the immunolabeled section with putative SCs labeled green, migrating right along the basement membrane to the transit-amplifying cell region and then upwards to surface to the terminally-differentiated cell region.

Figure 1A shows a section of human limbus and peripheral cornea that have been immunolabeled with the corneal SC marker K15 (green); the basal lamina is identifiable by immunolabeling with laminin (red), and the nuclei (blue) are stained with DAPI. Thus, one can visualize the three putative regions of interest: the keratin 15-positive SCs in the limbal region, the keratin 15-negative TA cells further along the basement membrane in the cornea, and the TD cells that are more superficial. A schematic of this proposed tissue architecture is shown in Figure 1B.

**Figure 2:**
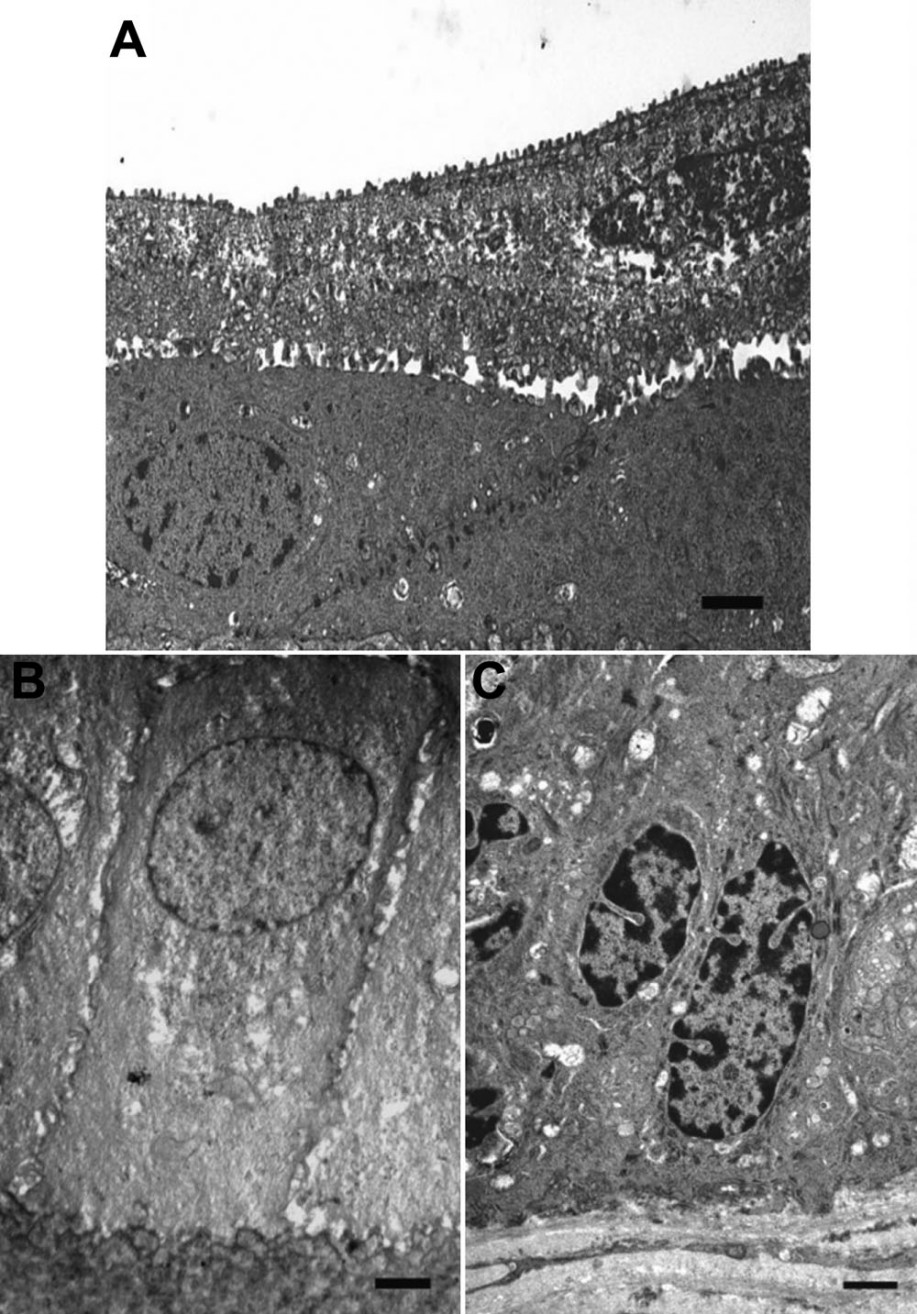
Ultrastructural analysis of cells types in human corneal epithelium. **A**: Transmission electron micrograph (TEM) of terminally-differentiated corneal epithelial cells. Cells on the surface were highly differentiated squamous cells with the most superficial being highly vesiculated with apoptotic nuclei and in the process of desquamating (scale bar=1 μm). **B**: TEM of the transit-amplifying corneal epithelial cells. These basal cells are large columnar cells and contain large round nuclei with diffuse chromatin (scale bar=1 μm). **C**: TEM of basal limbal stem cells. These cells are small with irregular nuclei that contain large amounts of condensed chromatin (scale bar=1 μm).

Examination of limbal and corneal epithelial cells by TEM shows that the limbal SCs, TA cells, and TD cells exhibit very different ultrastructural characteristics. TD cells consist of flat, plate-like, squamous, superficial cells (Figure 2A), some with apoptotic nuclei. TA cells are columnar in appearance with large round nuclei containing diffuse chromatin (Figure 2B). In contrast, limbal SCs are smaller in size and their cytoplasm is denser in comparison to basal corneal TA cells; in addition, their nuclei tend to be more irregular in shape, and a large proportion of the nuclear chromatin appears to be extremely dense (Figure 2C).

**Figure 3:**
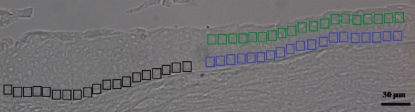
Unstained section of human cornea showing the putative regions where point spectra in transmission mode (4 cm^−1^ resolution, co-added for 256 scans) were acquired. Black apertures (8 μm×8 μm) designate the stem cell region, blue apertures the transit-amplifying cell region, and green apertures the terminally-differentiated cell region.

Based on such prior knowledge, three putative regions of the human limbus and cornea were designated for point spectrum analysis (8 μm×8 μm aperture) by SRS FTIR microspectroscopy (Figure 3). This included the SC region in the limbus (black apertures), the TA cells further along sitting on the basement membrane of the cornea (blue apertures), and the superficial TD cells (green apertures). Through each aperture, a biochemical-cell fingerprint in the form of a mid-IR spectrum was derived.

### Point spectral analysis of putative regions

**Figure 4:**
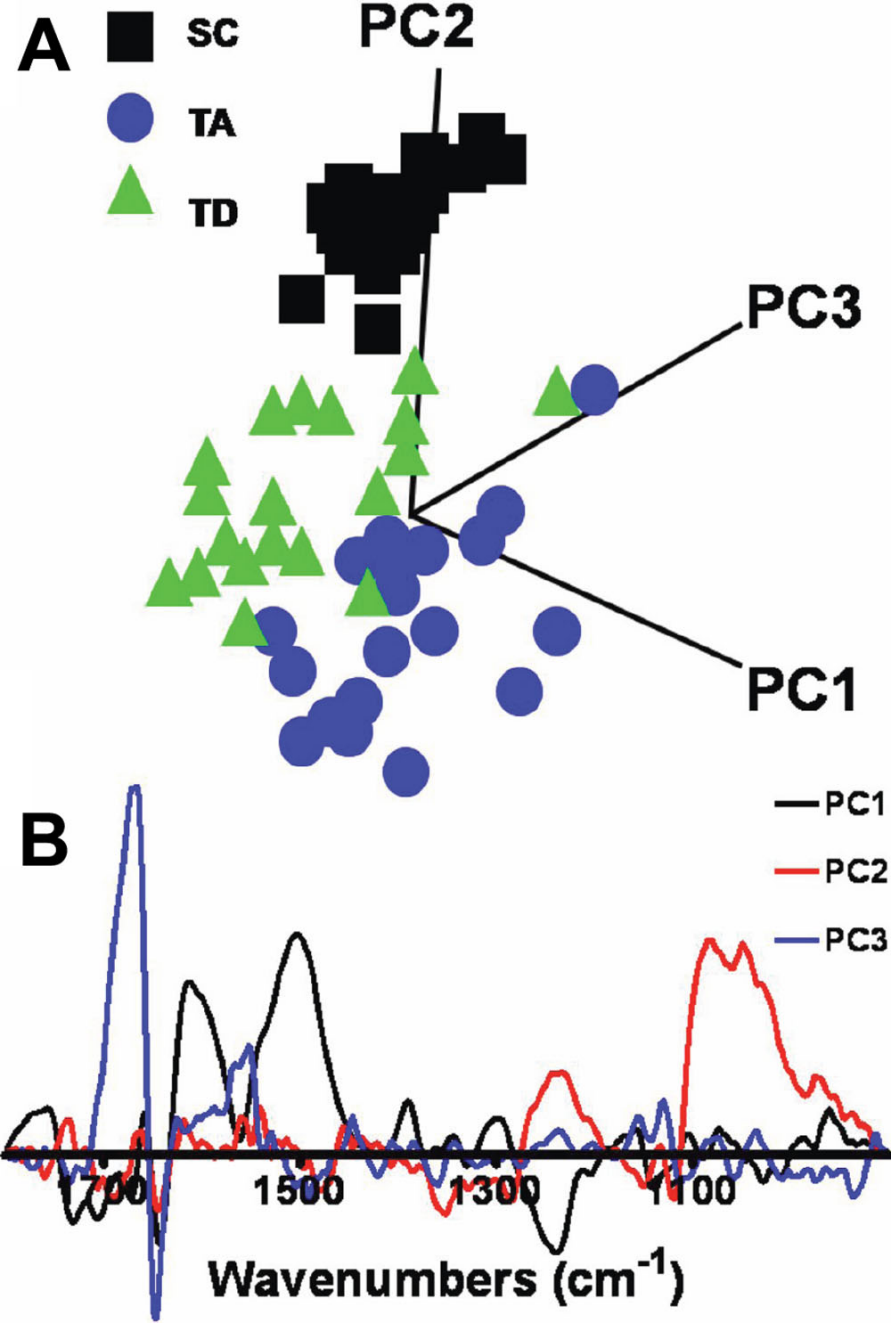
Principal component analysis of point mid-infrared spectra over the biologically relevant spectral region (1,800–900 cm^−1^) derived from the three putative regions (stem cell, transit-amplifying cell, and terminally-differentiated cell). **A**: The principal component analysis scores plots of stem cell (SC) versus transit-amplifying (TA) cell versus terminally differentiated (TD) cell shows good separation of the three cell populations with a small degree of overlap. **B**: The resultant loadings plot identifies the biomarker differences (i.e., discriminating wavenumbers) over the spectral range.

FTIR microspectroscopy generates highly complex data; it is difficult to identify important, often subtle, differences between different cell classes. Built on the assumption that variation implies information, PCA replaces the hundreds of spectral wavenumber variables by linear combinations (i.e., PCs), which seek to capture as much variability as possible. Thus, information in an entire IR spectrum is reduced to a single point, with coordinates on the one, two, or more PCs chosen as axes for the scores plots. In PCA, one acquires two types of information: cluster (scores) plots of class separation (Figure 4A) and loadings plots to identify the chief contributory variables (i.e., wavenumbers) that identify the class-specific information responsible for clustering (Figure 4B). Increasing spatial separation between points in a scores plot is proportional to the level of dissimilarity in absorbance spectra.

Figure 4A shows a scores plot for the spectral points associated with the SC, TA cell, and TD cell populations. Segregation between the three different cell classes was observed, with the points for SCs being tightly clustered and those for TA cells and TD cells being readily discriminated. Interestingly, in this view the TD-cell cluster was more closely aligned to SC points than those of TA cells. From the scores plot, PC2 is clearly the most important linear combination contributing toward separation of the three cell classes as it cuts through all three; loadings associated with PC3 would be most likely associated with intraclass variability. The loadings plot suggested that the discriminating wavenumbers associated with PC2 were primarily in the DNA/RNA region and included 1,040 cm^−1^ (C–O vibrations [DNA/lipids]), 1,080 cm^−1^ (symmetric phosphate stretching vibrations [ν_s_PO_2_^-^]), 1,107 cm^−1^ (sugar-phosphate vibrations), and 1,225 cm^−1^ (asymmetric phosphate stretching vibrations [ν_as_PO_2_^-^]; Figure 4B).

**Figure 5:**
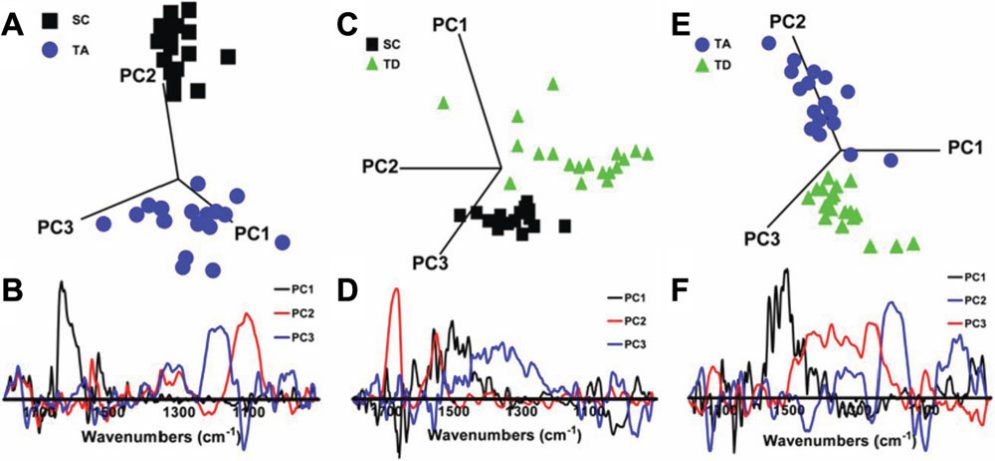
Two-class discrimination using principal component analysis over the biologically relevant spectral region (1,800–900 cm^−1^) derived from the three putative regions (stem cell [SC], transit-amplifying [TA] cell, and terminally-differentiated [TD] cell). Principal component analysis scores and loading plots of **A** and **B** SCs versus TA cells; **C** and **D** SCs versus TD cells; and **E** and **F** TA cells versus TD cells. Cell-specific clusters show good two-class discrimination (**A**,**C**,**E**), and the respective loadings plots (**B**,**D**,**F**) demonstrate major biomarker differences along chosen linear coordinates (i.e., principal components [PCs]).

Two-class discrimination was then used to identify the segregating biochemical entities between pairs of cell populations. Figure 5A shows the scores plot for SCs versus TA cells; perfect cluster separation was observed, pointing to PC2 being most responsible for this observation, and the corresponding loadings curve (Figure 5B) again identified 1,107 cm^−1^ and 1,120 cm^−1^ (C–O vibrations, [RNA]) as important. Changes in RNA cellular levels, whether they be translatable mRNA or noncoding or interfering transcripts, would be expected to be pivotal in the transition from SC to TA cells. On the scores plot showing separation of the SCs versus TD cells, the primary discriminating factor was PC1, whereas PC2 in this case would be expected to contribute mostly to intraclass variance (Figure 5C). Along PC1, the discriminating loadings were in the lipid/protein region, including 1,728 cm^−1^ (C=O stretch [lipids]), 1,555 cm^−1^ (C–N stretch NH bend [amide II]), 1,525 cm^−1^ and 1,400 cm^−1^; interestingly, 1,080 cm^−1^ was also highlighted (Figure 5D). Such observations would point to the differentiated functionality of the TD cells. From the scores plot of TA cells versus TD cells, PC1 was by far the most important discriminating factor between the two cell population clusters (Figure 5E). Along PC1, the corresponding loadings plot showed distinct loadings identifying discriminating wavenumbers throughout the IR spectrum; these included 1,728 cm^−1^, 1,400 cm^−1^, 1,225 cm^−1^, 1,107 cm^−1^, 1,080 cm^−1^, and 1,040 cm^−1^ (Figure 5F). Such observations suggest that lipid/protein and DNA/RNA conformational changes are both important in the transition from TA cells to TD cells.

### Spectral imaging

**Figure 6:**
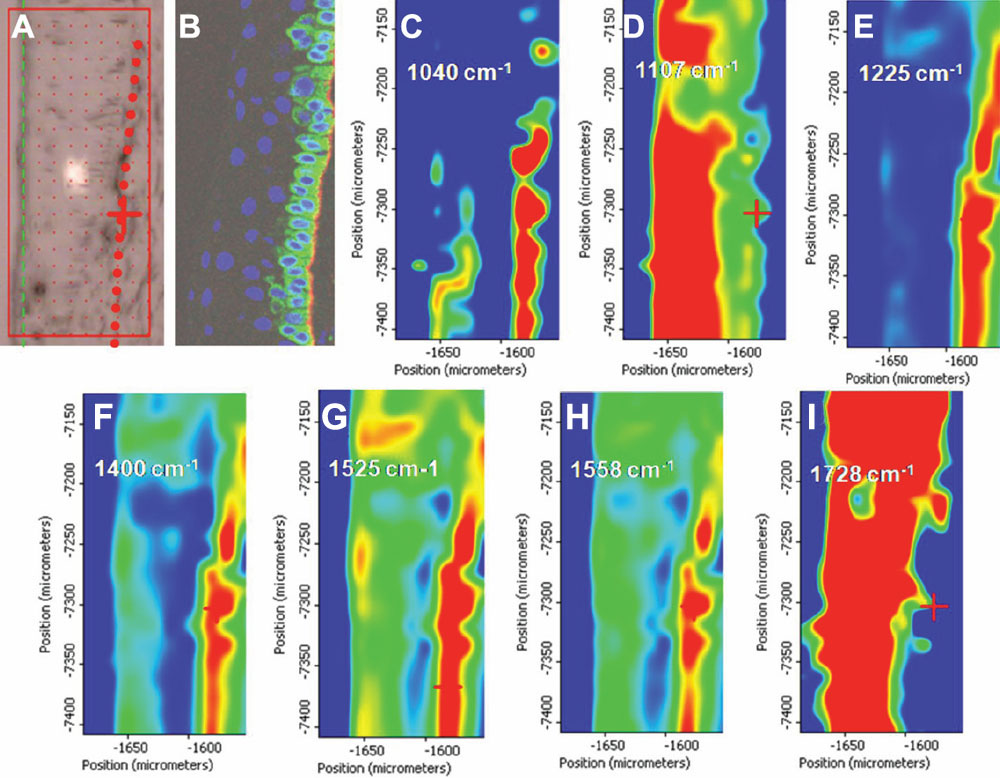
Spectral image maps of infrared absorbance at wavenumbers (cm^−1^) chosen to highlight the three putative regions (stem cell, transit-amplifying cell, and terminally-differentiated cell) of human cornea. **A**: An unstained cryosection of corneal limbus from which the maps were taken. The red rectangle shows the precise area from which the mid-infrared (IR) spectral map was acquired. The dotted red line shows the approximate location of the basal limbal stem cells. The green line indicates the superficial epithelium. **B**: The limbal region immunolabeled with a green fluorescent marker (keratin 15) for corneal SCs. The cell nuclei have been stained with DAPI (4',6-diamidino-2-phenylindole; blue), and the basement membrane has been immunolabeled for laminin (red). **C**: Mid-IR spectral map showing absorbance for 1,040 cm^−1^; **D**: mid-IR spectral map showing absorbance for 1,107 cm^−1^; **E**: mid-IR spectral map showing absorbance for 1,225 cm^−1^; **F**: mid-IR spectral map showing absorbance for 1,400 cm^−1^; **G**: mid-IR spectral map showing absorbance for 1,525 cm^−1^; **H**: mid-IR spectral map showing absorbance for 1,558 cm^−1^; and **I**: mid-IR spectral map showing absorbance for 1,728 cm^−1^.

IR spectral image maps allow one to track the spatial distribution of chemical entities based on levels of relative absorbance intensity at a chosen wavenumber in a pixel-by-pixel fashion, each pixel having an 8 μm×8 μm aperture. From this, one can acquire an image map in which the absorbance intensity is proportional to thermal color changes: blue (lowest intensity) < green < yellow < red (highest intensity). This allows the examination of whether particular chemical entities that had hitherto been identified as contributors to variance are differentially absorbed between the three putative regions designated as SC, TA cell, and TD cell (Figure 6). The spatial correlation for several wavenumbers was identified, and these wavenumbers were variously associated with the putative SC, TA cell, and TD cell regions. Figure 6A shows the cryosection from which the data were collected, while Figure 6B shows an equivalent cryosection which had been immunolabeled with the stem cell marker K15 (green), laminin (red) for the basement membrane, and the DAPI-stained nuclei (blue). Wavenumbers 1,040 cm^−1^ (Figure 6C), 1,225 cm^−1^ (Figure 6E), 1,400 cm^−1^ (Figure 6F), 1,525 cm^−1^ (Figure 6G), and 1,558 cm^−1^ (Figure 6H) clearly demarcated the SC region of the limbus. In contrast, the wavenumbers 1,107 cm^−1^ (Figure 6D) and 1,728 cm^−1^ (Figure 6I) were more strongly absorbed in the overlying TA cell and TD cell regions.

### Feature analysis based on wavenumber histograms

**Figure 7:**
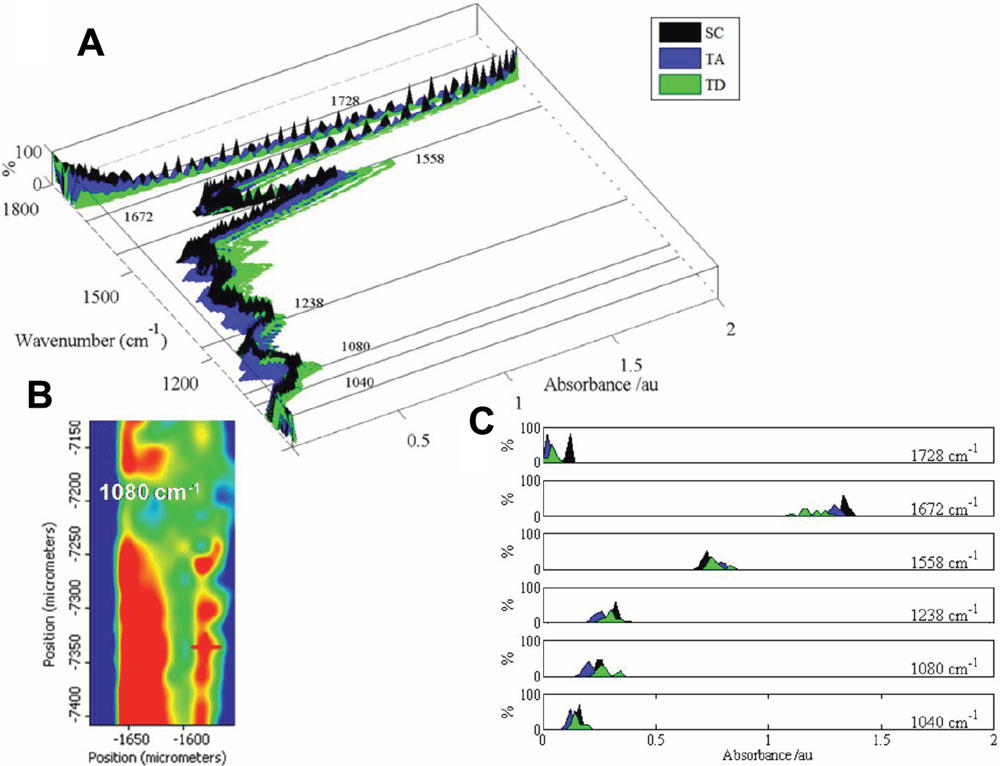
Distribution analysis of mid-infrared spectra derived from the three putative regions (stem cell, transit-amplifying cell, and terminally-differentiated cell) of human cornea. **A**: Percentage histograms for stem cells (SCs), transit-amplifying (TA) cells, and terminally differentiated (TD) cells×all wavenumbers put together in a three-dimensional form, where selected wavenumbers have been indicated. **B**: Mid-infrared spectral map showing absorbance for wavenumber 1,080 cm^−1^; and, **C**: specific histograms for selected wavenumbers suggested to be associated with marked class discrimination.

Each IR spectrum was acquired at 4 cm^−1^ resolution; histograms were computed as distribution percentages along 100 equal-length subintervals of the whole absorbance range [0, 2] (Figure 7A). Based on the identified biomarkers, this would allow one to visualize the intraclass distribution. Examining the wavenumbers previously highlighted, a spatial distribution in the absorbance intensity of various chemical entities was noted (e.g., 1,080 cm^−1^) (Figure 7B). Typically, SC spectra (black) showed less variability with all spectra concentrated around an average prototype. In contrast, TD spectra were the most widely distributed (Figure 7D). SC-associated IR spectra were completely distinguished and non-overlapping with TA cell and TD cell histograms with several wavenumbers, especially 1,728 cm^−1^.

## Discussion

Microspectroscopy has been used with some success in the identification and characterization of many cell types [29,30], e.g., distinguishing both malignant and premalignant changes in several different tissues [18-20,31,32] and identifying differences in bovine [1] and more recently in human SC and TA corneal epithelial cells [2]. Previously, such studies on human cornea only compared SC versus TA cells; in this study, we interrogated the entire tissue architecture for spectral imaging of the limbus and cornea. This was performed with more conventional analyses, using immunolabeling (Figure 1) and TEM (Figure 2) to confirm the spatial locations of corneal SCs, TA cells, and TD cells.

TEM illustrates just how different these cells are, in particular the basal SCs, which are small with unspecialized, small, irregular nuclei (Figure 2). Importantly, the nuclear chromatin appeared to be extremely dense and compact in comparison to those of the TA cells and TD cells. The TA cells are large columnar cells and contain large round nuclei with diffuse chromatin (Figure 2), pointing to actively dividing cells. Although the TD cells often appeared apoptotic with vesiculated nuclei, this is in agreement with other studies [33]. Taking into account these class distinctions with regards to cell type (Figure 3), SRS FTIR microspectroscopy coupled with multivariate analysis segregated IR spectra derived from SC, TA cell, and TD cell populations into discrete clusters (Figure 4). Loadings plots highlighted the discriminating wavenumbers across the entire spectral range (1,800–900 cm^−1^); observations associated with DNA/RNA alterations are in agreement with previous studies [3] and are not surprising when one observes ultrastructural differences in cell-specific nuclei. Additionally, the cytoplasm of the SCs, TA cells, and TD cells appear different, and there are major changes in the expression of many proteins, including the keratin pairs K3/K12, clusterin, aldehyde dehydrogenase, and the gap junction protein connexion; this could explain why there are major differences in the protein regions of IR spectra (1,800–1,480 cm^−1^) derived from different cell types.

In this investigation we set out to identify specific IR spectral biomarkers that would discriminate the SCs compared to the TA cells and TD cells. Multivariate analysis of derived point IR spectra derived from three putative regions (SC versus TA cell versus TD cell) points to wavenumbers differentially absorbed by the SCs in comparison to the TA cells or TD cells. Differentially absorbed wavenumbers include 1,040 cm^−1^, 1,080 cm^−1^, 1,107 cm^−1^, 1,225 cm^−1^, 1,400 cm^−1^, 1,525 cm^−1^ 1,558 cm^−1^, and 1,728 cm^−1^ (Figure 6 and Figure 7). Absorbance of the wavenumbers 1,525 cm^−1^ and 1,558 cm^−1^ are within the amide II band; N–H bending in proteins is associated with β-sheet conformation. Absorbance at 1,728 cm^−1^ (which is strongly lipid associated) occurs in the overlying TA cells and TD cells but not in the basal SCs; this wavenumber is associated with esters found within lipids and amino acid side chains (Figure 6). The absorbance at 1,400 cm^−1^ by the SC region is associated with C–O stretching in carbohydrates derived from amino acid side chains or lipids. Absorbance at 1,040 cm^−1^ probably arises from vibrations associated with deoxyribose and has been observed in structural modifications in DNA associated with cancer formation, an interesting possible link between stem cells and cancer cells [34]. For instance, adenosine diphosphate has an absorbance maximum at 1107 cm^−1^ [35].

Perhaps the most exciting of all is that the wavenumbers most associated with DNA conformation (1,225 cm^−1^ and 1,080 cm^−1^) appeared to distinguish corneal SCs, and these have been defined as SC markers in other tissues [33]. The fact that these markers are found in the SCs of very diverse tissues is of enormous significance. These findings show that we are able to identify classes of molecules but not yet definitively identify the specific molecules linked with SCs or SC differentiation. The next step would be to collect mid-IR spectra from specific organelles, most obviously the nucleus [36,37]. Furthermore, collection of spectra from live cells [23] might provide better characterization of the differentiation process [29,38]; an important additional tool for this purpose will be the application of Raman spectroscopy [30].

In conclusion, our study has demonstrated that SRS FTIR microspectroscopy in conjunction with subsequent computational analysis is able to identify discriminating biomarkers of SCs, TA cells, and TD cells in human cornea. Spectral imaging demonstrates the region-specific location (SC versus TA cell versus TD cell) of such biomarkers, highlighting the usefulness of this approach in situ. Significantly, these wavenumbers might be common to SCs of different tissues [39,40]. The nondestructive application of FTIR microspectroscopy to characterize biomarkers of SCs has the potential to be a powerful adjunct to more conventional approaches, such as immunolabeling or TEM.
